# Subclinical hypomania, psychiatric and neurodevelopmental diagnoses: phenotypic and aetiological overlap

**DOI:** 10.1111/jcpp.70045

**Published:** 2025-09-06

**Authors:** Georgina M. Hosang, Miriam I. Martini, Angelica Ronald, Henrik Larsson, Sebastian Lundström, Paul Lichtenstein, Mark J. Taylor

**Affiliations:** ^1^ Centre for Psychiatry and Mental Health, Wolfson Institute of Population Health, Barts and the London School of Medicine and Dentistry, Queen Mary University of London London UK; ^2^ Department of Medical Epidemiology and Biostatistics Karolinska Institutet Stockholm Sweden; ^3^ School of Psychology, Faculty of Health and Medical Sciences University of Surrey Guildford UK; ^4^ Department of Medical Sciences Örebro University Örebro Sweden; ^5^ Gillberg Neuropsychiatry Centre University of Gothenburg Gothenburg Sweden; ^6^ Centre for Ethics, Law and Mental Health University of Gothenburg Gothenburg Sweden

**Keywords:** Hypomania, psychiatric illnesses, neurodevelopmental conditions, twin study, genetic

## Abstract

**Background:**

Subclinical hypomanic symptoms are fairly common in the general population but are linked to psychiatric and neurodevelopmental conditions. However, the genetic and environmental origins of these associations are unclear. This twin study examined the phenotypic and aetiological associations between subclinical hypomania and psychiatric and neurodevelopmental diagnoses.

**Methods:**

Participants were 4,932 twin pairs from the Child and Adolescent Twin Study in Sweden. Hypomanic symptoms were assessed using the parent‐rated Mood Disorders Questionnaire when the twins were aged 18. Specialist diagnoses of 14 conditions and symptoms were ascertained from Swedish population registries. Phenotypic associations between hypomania and these conditions/symptoms were investigated, and their aetiological overlap was examined using the twin method.

**Results:**

Subclinical hypomania was significantly associated with all 14 diagnoses. The highest odds were for psychotic disorders (odds ratio [OR] = 1.48, 95% confidence intervals [CI] = 1.33–1.64, *p* < .001). The genetic correlations between subclinical hypomania and these diagnoses ranged from 0.12 (95% CI: 0.04–0.33) for eating disorders (other than anorexia) to 0.58 (95% CI: 0.28–1.00) for drug misuse disorders. The nonshared environmental correlations were highest for psychotic disorders (0.52, 95% CI: −0.02 to 0.92) and lowest for body dissatisfaction (0.04, 95% CI: −0.01 to 0.08). For bipolar disorder, psychotic disorders, and attention deficit hyperactivity disorder, genetic, and nonshared environmental correlations with subclinical hypomania were of a similar magnitude.

**Conclusions:**

The association between subclinical hypomania and the diagnosis of multiple psychiatric phenotypes highlights its important role in the developmental pathway to clinical disorders, its complex origins, and that it may represent a quantitative trait for various psychiatric phenotypes.

## Introduction

Mania and hypomania (periods of increased energy, elation and irritability) characterise bipolar disorder, an enduring and serious psychiatric illness that affects up to 2% of the population (APA, 2013) that typically manifests in adolescence or early adulthood (Merikangas et al., 2011; Perlis et al., 2009). Subclinical levels of hypomania, in contrast, are more common, with up to 10% of adolescents categorised at high risk of bipolar disorder based on the number, duration, and impact of these symptoms (Hosang, Cardno, Freeman, & Ronald, 2017). The prevalence of subclinical hypomania is of great clinical concern given that youths reporting such symptoms are at increased odds of experiencing subsequent manic or hypomanic episodes and BD onset (Axelson et al., 2015). Subclinical hypomania has also been linked to other negative consequences, such as suicidal behaviours, reduced life satisfaction, and co‐occurring psychopathological symptoms (e.g. anxiety symptoms) (Hosang et al., 2017; Merikangas et al., 2011). Up to a third of people experiencing subclinical hypomania report using mental health services (Merikangas et al., 2011). Therefore, gaining a greater understanding of hypomania will inform efforts to reduce these negative correlates and consequences.

There is evidence that subclinical hypomania (especially when it manifests in childhood or adolescence) may be on the path between subclinical symptoms and clinical disorders, and thus an important target for prevention and intervention strategies (González‐Calvo et al., 2024). For instance, one study conducted a 15‐year follow‐up of adolescents reporting subclinical hypomania and found higher rates of various psychiatric disorders compared with controls; these included major depression, anxiety disorders, obsessive compulsive disorder [OCD], personality disorders, and drug and alcohol abuse (Päären et al., 2014). The results from the World Mental Health Initiative using data from 11 countries found that lifetime psychiatric comorbidities were highly prevalent (~69%) among people with ‘subthreshold’ or subclinical hypomania, the most common of which were panic attacks (41.7%), alcohol abuse (28.2%) and separation anxiety disorder (23.3%) (Merikangas et al., 2011). However, some psychiatric disorders (e.g., psychotic and eating disorders) have not been fully investigated in these studies, likely due to their low prevalence (Päären et al., 2014).

To build a comprehensive picture of the clinical correlates and outcomes of subclinical hypomania, it is important to consider conditions and behaviours which commonly co‐occur with psychopathology and typically require clinical attention. Sleep disturbances (e.g. difficulty in initiating or maintaining sleep) are hallmarks of various psychiatric disorders (Freeman, Sheaves, Waite, Harvey, & Harrison, 2020) and are linked to subclinical hypomanic symptoms (Hosang et al., 2017). But questions remain about more severe sleep conditions (e.g. insomnia) which require clinical interventions (e.g. medication) and warrant further investigation in the context of subclinical hypomania. Self‐harm is associated with increased risk of suicide attempts and a major cause of hospital admission (Carroll, Metcalfe, & Gunnell, 2014). Self‐harm is highly prevalent in psychiatric disorders (Singhal, Ross, Seminog, Hawton, & Goldacre, 2014), but less is known about how it relates to subclinical hypomania. The results of one study provide preliminary evidence of a weak relationship between *self‐reported* self‐harm behaviours and hypomanic symptoms (Fang et al., 2022), but need replicating especially using more objective measures of self‐harm to reduce reporting bias.

Hypomania may also be a psychopathological consequence or correlate of neurodevelopmental conditions (APA, 2013). For instance, the diagnosis of Attention Deficit/Hyperactivity Disorder [ADHD] and autism has been associated with elevated levels of subclinical hypomania at ages 15 and 18 (Hosang, Lichtenstein, Ronald, Lundström, & Taylor, 2019; Taylor et al., 2022).

Beyond associations, it is crucial to consider the factors that may explain the overlap between subclinical hypomania and clinical diagnoses and symptoms. The results of such work help build a clearer picture of the unique and shared aetiology of hypomania and such phenotypes. For instance, twin analyses reveal that genetic factors explain 72% of the phenotypic correlation between subclinical adolescent hypomania and bipolar disorder, with nonshared environmental factors making a smaller contribution (28%) (Hosang et al., 2022). At the symptom level, twin studies focused on neurodevelopmental traits/conditions report that up to 29% of the variance in ADHD traits and 6%–9% of autistic traits can be explained by genetic influences shared with adolescent hypomania (Hosang et al., 2019; Taylor et al., 2022). More research is warranted, focused on other conditions that are known to be strongly (phenotypically) associated with subclinical hypomania, such as anxiety disorders and drug and alcohol misuse.

Initial findings indicate the importance of stratifying the results by sex. For instance, higher heritability estimates for hypomania among males (59%) compared with females (29%) have been reported (Hosang et al., 2022). But a twin study reported that a higher proportion of the variance in hypomania was explained by shared genetic influences with ADHD among females (28%) compared with males (14%) (Hosang et al., 2019). This area requires further investigation, extended to other conditions.

This is the first study to explore the association between subclinical hypomanic symptoms in youths (at age 18) with the diagnosis of psychiatric, neurodevelopmental conditions and related symptoms (up to age 24), exploring the degree of genetic and environmental correlations between these phenotypes drawing on population health registry and twin data. This study will address two specific aims. First, the relationship between subclinical hypomania and the diagnosis of 14 such conditions and symptoms will be examined. It is hypothesised that significant associations will be observed between subclinical hypomania and the diagnosis of all conditions and symptoms investigated. The second aim is to investigate the extent to which genetic and environmental factors for subclinical hypomania are associated with the 14 diagnoses. It is anticipated that subclinical hypomania will have the greatest aetiological overlaps with bipolar disorder.

## Methods

### Sample

Each year since 2004, families of Swedish twins aged 9 years are invited to participate in the Child and Adolescent Twin Study in Sweden [CATSS] (Anckarsäter et al., 2011) and are followed up at age 18 years (Anckarsäter et al., 2011). At age 18, data on parent‐reported hypomanic symptoms and official diagnoses of the target disorders were available for 4,932 twin pairs from CATSS. Parents and twins provided written informed consent before participation. CATSS was approved by the Regional Ethical Review Board in Stockholm on 31^st^ December 2016 (ref: 2016/2135‐31).

### Materials

Hypomanic symptoms were measured using the parent‐report Mood Disorder Questionnaire [MDQ] (Wagner et al., 2006) when the twins were 18 years old. The MDQ's 13 yes/no items relate to the presence of symptoms based on the Diagnostic and Statistical Manual for Mental Disorders Fourth Edition [DSM‐IV] criteria for a hypomanic or manic episode (American Psychiatric Association, 1994; Wagner et al., 2006). Additional items enquire whether symptoms occur during the same period (episode) and their impact on functioning (impairment). The parent‐rated MDQ has good sensitivity (0.72) and specificity (0.81) in identifying adolescent bipolar disorder (Wagner et al., 2006; Youngstrom, Freeman, & McKeown Jenkins, 2009). Hypomanic symptoms were used on a continuous (total number of symptoms) and on a dichotomous (categorised as ‘high‐risk’ of bipolar disorder assigned a value of ‘1’ or ‘0’ for the comparison group) scale. Individuals were categorised as ‘high‐risk’ of bipolar disorder if at least 7 MDQ items were endorsed, clustered in the same period, and caused moderate/severe impairment (Hirschfeld et al., 2000).

Lifetime psychiatric and neurodevelopmental diagnoses up to age 24 were identified using the Swedish National Patient Register (Ludvigsson et al., 2011) which documents all specialist inpatient care since 1987 and outpatient care since 2001 delivered to residents of Sweden. The International Statistical Classification of Diseases and Related Health Problems, Tenth Revision [ICD‐10] codes recorded on the NPR were extracted for 14 conditions (bipolar disorder, depressive disorders, anxiety disorders, OCD, psychotic disorders, anorexia nervosa, other eating disorders, ADHD, autism, sleep disturbances, borderline personality disorder, self‐harm, alcohol, and substance use disorders). For bipolar, psychotic, and sleep disorders, additional individuals were identified through dispensed prescriptions of medication in the Prescribed Drug Register (Table S1) (Wettermark et al., 2007).

### Statistical procedures

Continuous hypomania data were log‐transformed since they were positively skewed. Subclinical hypomanic symptoms were used in two ways: (a) the number of hypomanic symptoms (continuous variable) and (b) high‐risk of bipolar disorder categorisation (dichotomous variable) where 7 MDQ items were endorsed that clustered in the same period and caused moderate/severe impairment. For the latter, raw rather than log‐transformed hypomania scores were used, given the categorisation of this group is based on published recommendations. The sample was categorised into high‐risk for bipolar disorder and comparison (remaining sample) groups based on the MDQ cut‐off described earlier. Logistic regression models implemented as generalised estimating equations [GEE] framework to account for the inclusion of related individuals were used to calculate the odds of each diagnosis associated with the number of hypomanic symptoms. The increase in odds for each outcome per unit increase in hypomania score was calculated. The odds of each diagnosis associated with being at a high risk for bipolar disorder were also calculated. Sex and birth year were controlled for in all analyses. GEE models were implemented in the drgee package of R version 4.3.1 (R Foundation) (Zetterqvist & Sjölander, 2015). A one‐sided *p* < .05 was considered statistically significant.

#### Twin analyses

The classic twin design was used to determine the degree to which genetic and environmental factors influence individual phenotypes and the degree they are shared between them. Variance in liability for each phenotype can be divided into additive genetic (A), shared (C; common to both twins and creating similarity) and nonshared (E, unique to each twin and creating difference and measurement error) environmental factors. This division is based on the assumption that identical or monozygotic [MZ] twins share 100% of their segregating DNA code compared with nonidentical or dizygotic [DZ] twins who share 50% on average. Higher MZ twin pair correlations compared with those of DZ twin pairs indicate genetic influences on a phenotype. A detailed description of the general twin design principles is described elsewhere (Knopik, Neiderhiser, Defries, & Plomin, 2017). A joint categorical‐continuous bivariate model was used to estimate the genetic and environmental correlations between each diagnosis and hypomania, and the degree to which overlapping genetic and environmental influences explained their association (Martin, Taylor, & Lichtenstein, 2018). Twin models were conducted in the OpenMx package for R (Neale et al., 2016).

## Results

A total of 4,932 twin pairs (1,517 MZ and 3,415 DZ twin pairs) were included in this study; descriptive statistics by sex are presented in Table 1. The mean hypomania score was 0.84 (*SD* = 1.83) with 1.2% of the sample being categorised as high‐risk for bipolar disorder based on the number and negative impact of their hypomanic symptoms. The prevalence of the 14 diagnoses ranged from 0.2% for psychotic disorders and borderline personality disorder to 3.0% for ADHD.

**Table 1:** Sample description

	Overall sample		Females		Males	
	*N*	%	*N*	%	*N*	(%)
Sample (twin pairs)	4,932		1,518	30.8	1,719	34.9
Zygosity (twin pairs)						
MZ	1,517	30.8	670	44.1	847	24.8
DZ same sex	1720	34.9	848	55.9	872	25.5
DZ opposite sex	1,695	34.4	–		–	
Phenotype						
MDQ score (mean, *SD*)	0.84	1.83	0.80	1.80	0.88	1.85
High risk for bipolar disorder (MDQ cut‐off^a^)	110	1.2	49	1.1	61	1.2
Bipolar disorder	34	0.3	7	0.1	27	0.5
Psychotic disorders	17	0.2	10	0.2	7	0.1
Depressive disorders	267	2.7	79	1.7	188	3.7
Anxiety disorders	77	0.8	35	0.7	42	0.8
Alcohol use disorders	155	1.6	70	1.5	85	1.7
Substance use disorders	201	2.0	97	2.1	104	2.0
Anorexia nervosa	76	0.8	7	0.1	69	1.3
Other eating disorders	98	1.0	9	0.2	89	1.7
Borderline personality disorder	17	0.2	0	0.0	17	0.3
Obsessive compulsive disorder	47	0.5	22	0.5	25	0.5
ADHD	300	3.0	191	4.0	109	2.1
Autism	39	0.4	25	0.5	14	0.3
Self‐harm	180	1.8	80	1.7	100	1.9
Sleep disorders and disturbances	188	1.9	56	1.2	132	2.6

ADHD, attention deficit/hyperactivity disorder; MDQ, Mood Disorders Questionnaire; %, percentage.

^a^ Mood Disorders Questionnaire score of at least seven with symptoms clustered together in the same period and moderate to severe problems (e.g. work and legal problems).

### Subclinical hypomania, psychiatric, neurodevelopmental conditions, and related symptoms

The associations between hypomania and the 14 diagnoses were examined using two approaches focusing on (a) the number of hypomanic symptoms and (b) high‐risk of bipolar disorder categorisation (seven MDQ items were endorsed that clustered in the same period and caused moderate/severe impairment).

Subclinical hypomanic symptoms were associated with increased odds of being diagnosed with all 14 conditions and symptoms (Table 2). The highest odds were for psychotic disorders (odds ratio [OR] = 1.48, 95% confidence intervals [CI] 1.33–1.64, *p* < .001), bipolar disorder (OR = 1.40, 95% CI: 1.29–1.52, *p* < .001), ADHD (OR = 1.34, 95% CI: 1.30–1.39, *p* < .001) and borderline personality disorder (OR = 1.34, 95% CI: 1.21–1.48, *p* < .001). Being at high risk for bipolar disorder was significantly associated with the diagnoses of 13 of the 14 diagnoses; the highest odds were observed for psychotic disorders (*N* = 17 (0.2%); OR = 41.22, 95% CI: 14.48–117.39, *p* < .001), bipolar disorder (*N* = 34 (0.3%); OR = 32.22, 95% CI: 13.54–76.63, *p* < .001) and borderline personality disorder (*N* = 17 (0.2%); OR = 18.66, 95% CI: 4.89–71.13, *p* < .001). Although those at high risk for bipolar disorder had over double the odds for being diagnosed with autism, this association was not significant (OR = 2.60, 95% CI: 0.35–19.28, *p* > .05).

**Table 2:** Phenotypic associations between hypomania with psychiatric, neurodevelopmental, and related conditions

	Hypomanic symptoms				High‐risk group	High‐risk for bipolar disorder		
	OR (95% CI)	Beta	*SE*	*p*		Comparison group	OR (95% CI)	*p*
Bipolar disorder	1.40 (1.29–1.52)	0.34	0.04	<.001	9 (8.2%)	24 (0.3%)	32.22 (13.54–76.63)	<.001
ADHD	1.34 (1.30–1.39)	0.29	0.02	<.001	31 (28.2%)	251 (2.7%)	15.46 (9.96–24.01)	<.001
Alcohol misuse disorders	1.22 (1.17–1.28)	0.20	0.02	<.001	11 (10%)	138 (1.5%)	6.71 (3.54–12.73)	<.001
Anorexia nervosa	1.19 (1.12–1.26)	0.17	0.03	<.001	<5^a^	73 (0.8%)	3.40 (1.08–10.65)	.04
Anxiety disorders	1.19 (1.13–1.26)	0.18	0.03	<.001	6 (5.5%)	69 (0.7%)	7.20 (3.04–17.07)	<.001
Autism	1.20 (1.12–1.30)	0.19	0.04	<.001	<5^a^	35 (0.4%)	2.60 (0.35–19.28)	.351
BPD	1.34 (1.21–1.48)	0.29	0.05	<.001	<5^a^	13 (0.1%)	18.66 (4.89–71.13)	<.001
Depression	1.26 (1.22–1.30)	0.23	0.02	<.001	22 (20%)	229 (2.5%)	9.64 (6.02–15.45)	<.001
Substance misuse disorders	1.27 (1.22–1.32)	0.24	0.02	<.001	21 (19.1%)	170 (1.8%)	11.99 (7.16–20.06)	<.001
Self‐harm	1.19 (1.14–1.25)	0.18	0.02	<.001	11 (10%)	164 (1.8%)	6.08 (3.19–11.57)	<.001
Sleep disturbances	1.25 (1.19–1.31)	0.22	0.02	<.001	20 (18.2%)	164 (1.8%)	11.96 (7.10–20.16)	<.001
OCD	1.19 (1.11–1.28)	0.17	0.04	<.001	<5^a^	44 (0.5%)	5.71 (1.73–18.84)	.004
Other eating disorders	1.15 (1.08–1.22)	0.14	0.03	<.001	<5^a^	91 (1%)	3.60 (1.30–10.00)	.014
Psychotic disorders	1.48 (1.33–1.64)	0.39	0.05	<.001	6 (5.5%)	11 (0.1%)	41.22 (14.48–117.39)	<.001

ADHD, attention deficit/hyperactivity disorder; BPD, borderline personality disorder; CI, confidence interval; OCD, obsessive compulsive disorder; OR, odds ratio; *p*, *p* value; SE, standard error.

^a^ Due to Swedish Twin Registry regulations around anonymity, we are unable to present frequencies below 5.

### Genetic and environmental correlations between subclinical hypomania and psychiatric, neurodevelopmental conditions, and related symptoms

Phenotypic and cross‐trait cross‐twin analyses for subclinical hypomania and each diagnosis are presented in Table 3. The cross‐trait cross‐twin correlations were higher for MZ compared with DZ twins, indicating genetic contribution to the covariance between subclinical hypomania and each diagnosis. ACE bivariate models' fit statistics are provided in Table S2. The results could not be calculated for self‐harm (nonsignificant heritability) and borderline personality disorder (low number of concordant twins).

**Table 3:** Cross‐trait cross‐twin correlations for hypomania and the diagnosed disorders

	Twin correlations		Cross‐trait cross‐twin correlations with hypomanic symptoms		
	MZ	DZ	rPH	MZ	DZ
	Correlation coefficient (95% CI)	Correlation coefficient (95% CI)	Correlation coefficient (95% CI)	Correlation coefficient (95% CI)	Correlation coefficient (95% CI)
Hypomanic symptoms	0.56 (0.52–0.59)	0.28 (0.24–0.31)	–	–	–
High‐risk for bipolar disorder	0.65 (0.26–0.86)	0.25 (−0.05–050)	–	–	–
Bipolar disorder	0.88 (0.74–0.95)	0.40 (0.13–0.62)	0.36 (0.27–0.44)	0.29 (0.15–0.41)	0.06 (−0.06–0.17)
ADHD	0.90 (0.87–0.93)	0.48 (0.43–0.54)	0.39 (0.35–0.43)	0.32 (0.27–0.38)	0.15 (0.10–0.20)
Alcohol misuse disorders	0.69 (0.58–0.78)	0.39 (0.28–0.50)	0.22 (0.16–0.27)	0.25 (0.17–0.33)	0.07 (0.01–0.14)
Anorexia nervosa	0.73 (0.56–0.84)	0.16 (−0.17–0.42)	0.18 (0.11–0.25)	0.02 (−0.08–0.13)	−0.06 (−0.16–0.04)
Anxiety disorders	0.56 (0.38–0.70)	0.14 (−0.10–0.34)	0.18 (0.10–0.26)	0.09 (−0.03–0.21)	0.01 (−0.08–0.10)
Autism	0.92 (0.85–0.97)	0.43 (0.27–0.56)	0.20 (0.09–0.31)	0.15 (0.02–0.28)	0.16 (0.04–0.28)
BPD	−0.92 (−1.00–0.72)	−0.99 (−1.00–0.59)	0.33 (0.21–0.45)	0.46 (0.29–0.61)	0.05 (−0.14–0.25)
Depression	0.69 (0.60–0.76)	0.34 (0.25–0.42)	0.28 (0.23–0.32)	0.21 (0.14–0.28)	0.10 (0.04–0.15)
Drug misuse disorders	0.65 (0.55–0.74)	0.41 (0.31–0.49)	0.28 (0.24–0.33)	0.25 (0.18–0.33)	0.09 (0.03–0.16)
Self‐harm	0.57 (0.45–0.67)	0.46 (0.37–0.54)	0.19 (0.13–0.24)	0.05 (−0.03–0.14)	0.10 (0.04–0.17)
Sleep difficulties	0.71 (0.63–0.78)	0.44 (0.36–0.51)	0.24 (0.19–0.29)	0.21 (0.13–0.29)	0.11 (0.05–0.17)
OCD	0.57 (0.31–0.76)	0.29 (0.03–0.50)	0.18 (0.09–0.27)	0.18 (0.05–0.31)	0.05 (−0.05–0.16)
Other eating disorders	0.78 (0.66–0.86)	−0.72 (−1.00–0.21)	0.12 (0.05–0.19)	0.09 (0.00–0.18)	0.00 (−0.09–0.10)
Psychotic disorders	0.83 (0.07–0.92)	−1.00 (−1.00–0.48)	0.42 (0.31–0.53)	0.32 (0.12–0.52)	0.03 (−0.14–0.20)

ADHD, attention deficit/hyperactivity disorder; BPD, borderline personality disorder; CI, confidence intervals; DZ, dizygotic twins; MZ, monozygotic twins; OCD, obsessive compulsive disorder; rPH, phenotypic associations.

**Figure 1:**
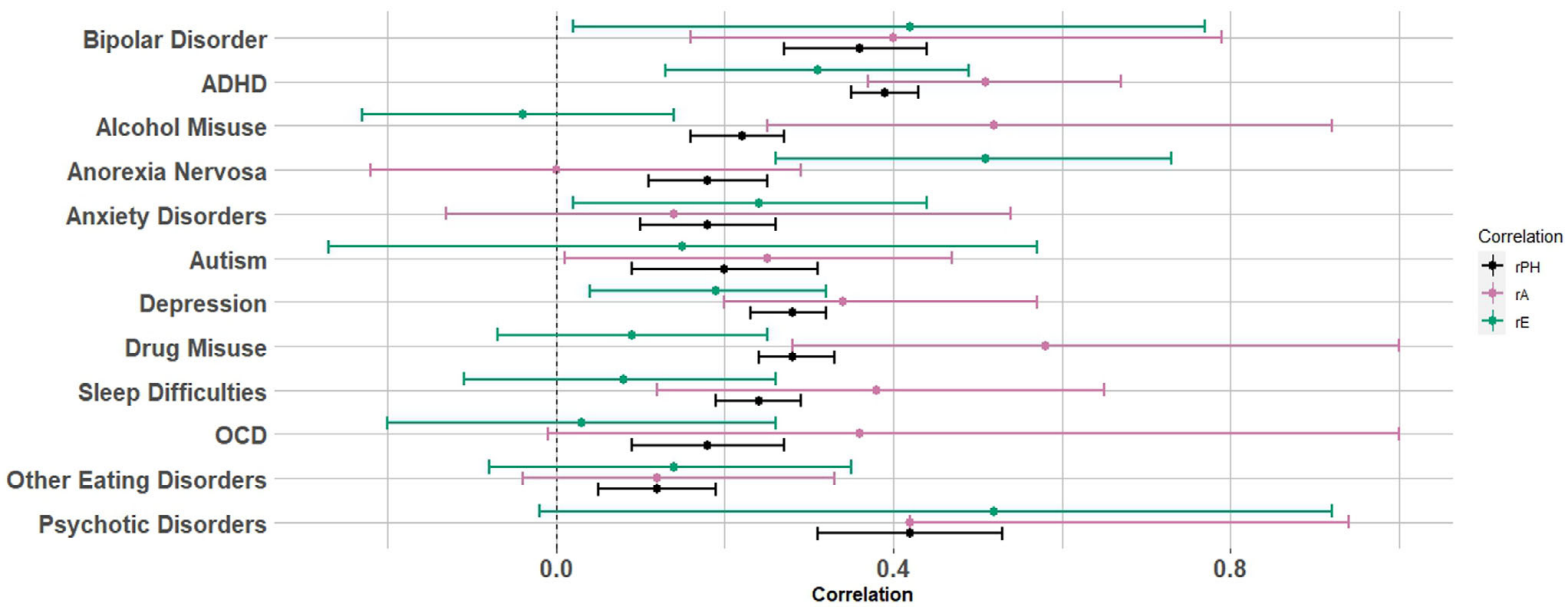
Phenotypic, genetic, and environmental correlations between subclinical hypomanic symptoms with psychiatric, neurodevelopmental, and related conditions. ADHD, attention deficit/hyperactivity disorder; BPD, borderline personality disorder; OCD, obsessive compulsive disorder; rA, genetic correlations; rE, nonshared environmental correlations; rPH, phenotypic associations. C (shared environmental influences) was close to 0 for each phenotype; therefore, rC could not be meaningfully calculated

The genetic correlations between subclinical hypomania and the remaining 12 diagnoses ranged from 0.00 (95% CI: −0.22 to 0.29) for anorexia nervosa to 0.58 (95% CI: 0.28–1.00) for drug misuse disorders (Table 4 and Figure 1). The highest genetic correlations observed also included alcohol misuse disorders (0.52, 95% CI: 0.25–0.92) and ADHD (0.51, 95% CI: 0.37–0.67). The nonshared environmental correlations varied between 0.52 (95% CI: −0.02 to 0.92) for psychotic disorders and − 0.04 (95% CI: −0.23 to 0.14) for alcohol misuse disorders. The highest nonshared environmental correlations included anorexia nervosa (0.51, 95% CI: 0.26–0.73) and bipolar disorder (0.42, 95% CI: 0.02–0.77).

**Table 4:** Genetic and environmental correlations between subclinical hypomania and clinical disorders

	Genetic correlations (rA; 95% CI)	Shared environmental correlations (rC; 95% CI)^a^	Nonshared environmental correlations (rE; 95% CI)	Proportion of covariation with hypomania explained by each aetiological component		
				A	C	E
Bipolar disorder	0.40 (0.16–0.79)	−1.00 (−1.00–1.00)	0.42 (0.02–0.77)	0.75	−0.05	0.30
ADHD	0.51 (0.37–0.67)	−1.00 (−1.00–1.00)	0.31 (0.13–0.49)	0.88	−0.05	0.17
Alcohol misuse disorders	0.52 (0.25–0.92)	−1.00 (−1.00–1.00)	−0.04 (−0.23–0.14)	1.28	−0.21	−0.08
Anorexia nervosa	0.00 (−0.22–0.29)	−1.00 (−1.00–0.97)	0.51 (0.26–0.73)	0.01	−0.04	1.03
Anxiety disorders	0.14 (−0.13–0.54)	−1.00 (−1.00–1.00)	0.24 (0.02–0.44)	0.4	−0.01	0.61
Autism	0.25 (0.01–0.47)	1.00 (−1.00–1.00)	0.15 (−0.27–0.57)	0.82	0.04	0.14
Depression	0.34 (0.20–0.57)	−1.00 (−1.00–1.00)	0.19 (0.04–0.32)	0.76	−0.01	0.25
Drug misuse disorders	0.58 (0.28–1.00)	−1.00 (−1.00–1.00)	0.09 (−0.07–0.25)	1.03	−0.16	0.12
Sleep difficulties	0.38 (0.12–0.65)	1.00 (−1.00–1.00)	0.08 (−0.11–0.26)	0.84	0.05	0.12
OCD	0.36 (−0.01–1.00)	−1.00 (−1.00–1.00)	0.03 (−0.20–0.26)	1.05	−0.11	0.06
Other eating disorders	0.12 (−0.04–0.33)	−1.00 (−1.00–1.00)	0.14 (−0.08–0.35)	0.62	0	0.38
Psychotic disorders	0.42 (0,42–0.94)	−1.00 (−1.00–1.00)	0.52 (−0.02–0.92)	0.65	−0.01	0.36

A, additive genetic influences; ADHD, attention deficit/hyperactivity disorder; C, shared environmental factors; CI, confidence intervals; E, nonshared environmental influences; OCD, obsessive compulsive disorder.

^a^ C (shared environmental influences) was close to 0 for each phenotype; therefore, rC could not be meaningfully calculated.

Some disorders had moderate genetic and nonshared environmental correlations, showing evidence of overall aetiological overlap with subclinical hypomania. First, the genetic (0.42, 95% CI: 0.42–0.94) and nonshared environmental (0.52, 95% CI: −0.02 to 0.92) correlation between psychotic disorders and subclinical hypomania was among the highest. Second, bipolar disorder had a genetic correlation of 0.40 (95% CI: 0.16–0.79) and nonshared environmental correlation of 0.42 (95% CI: 0.02–0.77) with subclinical hypomania. Finally, the aetiology between ADHD and subclinical hypomania was substantially correlated, with a genetic correlation of 0.51 (95% CI: 0.57–0.67) and nonshared environmental correlation of 0.31 (95% CI: 0.13–0.49).

Moreover, the phenotypic correlations with subclinical hypomania that had the highest proportions explained by genetic factors were with alcohol misuse (1.28), OCD (1.05), and drug use (1.03) (see Table 4). Whereas anorexia nervosa (1.03), anxiety (0.61) and other eating disorders (0.38) phenotypic correlations with subclinical hypomania had the highest proportions attributed to unique environmental influences. The proportions for shared environmental influences were either negative or negligible.

## Discussion

To the best of our knowledge, this study explored for the first time the associations between subclinical hypomania and the diagnosis of various conditions (e.g. eating disorders) using population registry data, as well as replicating some initial findings for related symptoms (sleep disturbances and self‐harm). This is the first study to explore the *aetiological overlap* between subclinical hypomania and the diagnosis of numerous psychiatric and neurodevelopmental conditions and related symptoms using twin data. Our results showed that subclinical hypomania was significantly associated with all diagnoses investigated. The strongest relationships were observed for subclinical hypomania and bipolar disorder, psychotic disorders, ADHD, and borderline personality disorder diagnoses. The results of the twin analyses revealed aetiological overlap between subclinical hypomania and 12 diagnoses. The highest genetic correlations were observed for subclinical hypomania with ADHD, drug and alcohol misuse disorders. Subclinical hypomania had the highest nonshared environmental correlations with psychotic disorders, anorexia nervosa, and bipolar disorder. Interestingly, both moderate genetic and environmental overlaps were observed with subclinical hypomania, bipolar disorder, psychotic disorders, and ADHD diagnoses, suggesting an overall shared aetiology not just in one domain (e.g. genetic).

This investigation provides novel contributions to the field by exploring and finding significant associations between adolescent subclinical hypomanic symptoms and the diagnosis of borderline personality disorders, eating disorders, and sleep difficulties for the first time, using population health registry data. In line with previous research, significant relationships between subclinical hypomania and the other conditions were also detected, especially psychotic disorders, bipolar disorder, and ADHD (Merikangas et al., 2011; Päären et al., 2014). The results highlight the importance of subclinical hypomania in the path between symptoms and psychiatric disorders, suggesting that these symptoms, even at a subclinical level, are worthy of intervention efforts.

This is the first study to investigate the *aetiological overlap* between subclinical hypomania and the diagnosis of psychiatric and neurodevelopmental conditions and symptoms using twin data. Our novel findings mirror the results reported for bipolar disorder, showing high genetic correlations with ADHD, alcohol, and substance use (Mullins et al., 2021; Song et al., 2015). We found the highest nonshared environmental correlations between subclinical hypomania and psychotic disorders, anorexia nervosa, and bipolar disorder. However, the results of a bipolar disorder adoption study revealed that the phenotypic correlation between drug abuse, anxiety, and personality disorders was largely explained by nonshared environmental factors (Song et al., 2015). These disparate findings could be attributed to our focus on subclinical hypomania rather than bipolar disorder. The aetiological overlap between subclinical hypomania and diverse psychiatric phenotypes demonstrates its complex genetic and environmental architecture, but also supports hierarchical models of psychopathology that recognise underlying shared susceptibility to psychopathology termed the ‘p factor’ alongside disorder‐specific vulnerabilities (Caspi et al., 2014; Ronald, 2019). Future studies should explore the aetiological overlap between subclinical hypomania and the ‘p’ factor.

The other noteworthy finding from this investigation is that several phenotypes showed notable overlap with subclinical hypomania in terms of both genetic and nonshared environmental influences, suggesting a shared aetiology overall, not for just one domain (e.g. genetic vs. nonshared environmental correlations). This was found for bipolar disorder, psychotic disorders, and ADHD. The results pertaining to hypomania and bipolar disorder provide further support to the postulation that hypomania represents a phenotypic continuum or quantitative trait of bipolar disorder (González‐Calvo et al., 2024; Hosang et al., 2022). However, the aetiological link between subclinical hypomania and other diagnoses (e.g. psychotic disorders) suggests that phenotypic continuums for clinical conditions are complicated and likely to involve multiple symptom dimensions. Identification of specific genetic and environmental factors shared between these phenotypes would also be informative and should be the focus of future investigations (Gonzalez‐Calvo et al., 2024; Hosang et al., 2024; Manoli, Wright, Shakoor, Fisher, & Hosang, 2023).

Our findings have important clinical and research implications. The significant associations between subclinical hypomania and the diagnosis of various psychiatric phenotypes that typically manifest in adolescence and adulthood (e.g. psychotic disorders) (Caspi et al., 2020) indicate that the early presentations of psychiatric illnesses may include hypomania. This suggests that hypomania should be considered a crucial component of developmental models of psychopathology and a prevention/intervention target to avoid progression to clinical disorders (González‐Calvo et al., 2024). However, subclinical hypomania may be a possible outcome or correlate of some psychiatric and neurodevelopmental conditions. For instance, we found that neurodevelopmental conditions (autism and ADHD), which manifest in early childhood (APA, 2013), were also significantly linked to subclinical hypomania at age 18. Moreover, some psychiatric conditions may have emerged prior to the presentation of hypomania at 18 years; depression and anxiety disorders are among some of the most common childhood mental health diagnoses (Kieling et al., 2024).

Subclinical hypomania's aetiological overlap with diverse diagnoses demonstrates the complexity of its origins and directs future studies designed to identify specific genetic and/or environmental influences. It is noteworthy that none of the genetic or nonshared environmental correlations between subclinical hypomania and the diagnoses investigated were indicating that there are hypomania‐specific aetiological factors that need to be identified by future research.

### Methodological considerations

The current investigation has various strengths, including utilisation of parent‐rated hypomanic symptoms from a psychometrically robust tool and specialist diagnoses from population registries. But some limitations should be considered when interpreting the findings. First, the diagnoses were taken from specialist services and do not capture illnesses commonly managed and treated in primary care (e.g. depressive and anxiety disorders) (Malhi & Mann, 2018; Szuhany & Simon, 2022). This may explain the moderate associations and aetiological overlap between subclinical hypomania and anxiety and depressive disorders. However, this is only relevant for depression and anxiety diagnoses after the age of 18 since *childhood* diagnoses of these disorders are captured by specialist services. Future studies should consider diagnoses made at all levels of care for a more comprehensive investigation of the relationship between clinical disorders and subclinical hypomania.

Second, the diagnosis identification strategy not only included the official diagnoses but also medication prescriptions to record clinical symptoms requiring treatment. This approach was undertaken for bipolar disorder (included lithium prescription), psychotic disorders (included clozapine) and sleep disturbances (included Melatonin, Zopiclone, Zolpidem, or Zaleplon prescriptions). This approach has been adopted by previous studies to comprehensively capture those affected by the disorder of interest and overcome diagnostic biases (Hosang et al., 2022; Martini et al., 2022). However, using lithium prescriptions may mean some individuals may be misclassified as having bipolar disorder since this medication is also used for treatment‐resistant depression (Uses, 1999). Thirdly, the low number of diagnoses for some disorders (e.g. psychotic disorders) means that the results should be interpreted cautiously. Fourth, it is possible that the genetic and environmental correlations between subclinical hypomania and the selected diagnoses may have varied by sex but were not examined in the current investigation. One study found that a greater proportion of hypomania's variance was attributable to shared genetic factors with ADHD among females (28%) compared with males (14%) (Hosang et al., 2019). Future research would benefit from exploring sex differences in the shared aetiology between subclinical hypomania and clinical diagnoses. Fifth, most ACE models fitted the data significantly worse than the saturated model. While this can commonly occur in twin studies based on large samples, it does mean that our results should be interpreted considering this caveat. Finally, the diagnoses were obtained when the participants were aged up to 24 years, which does not cover the full risk period of manifestation/diagnosis of various disorders (e.g. psychotic disorders and bipolar disorder) which means our results may be especially relevant for those that experience early onset or receive timely diagnosis (McGrath et al., 2023). Future studies should focus on psychiatric diagnoses at an older age when participants have passed through the risk period, to more accurately establish their link with subclinical hypomania.

## Conclusion

To the best of our knowledge, this is the first study to investigate the association and aetiological overlap between subclinical hypomania and various psychiatric and neurodevelopmental conditions and symptoms. We found significant relationships with all 14 diagnoses investigated; the strongest associations detected were with psychotic disorders, bipolar disorder, and ADHD. Substantial genetic or environmental correlations were observed between subclinical hypomania and the conditions investigated. However, for bipolar disorder, psychotic disorders, and ADHD, both the genetic and environmental correlations were of moderate magnitude with subclinical hypomania, suggesting a shared aetiology overall. Taken together, these findings indicate that hypomania is a critical component of a developmental psychopathology model and should be considered as part of the early presentations of psychiatric disorders. Moreover, its aetiological overlap with various disorders demonstrates the complex model of causation for hypomania. For those disorders where both moderate genetic and environmental correlations were observed with hypomania, it suggests that it may be a quantitative trait for several clinical disorders.

## Ethics statement

Parents and twins provided written informed consent before participation. CATSS was approved by the Regional Ethical Review Board in Stockholm on 31st December 2016 (ref: 2016/2135‐31).

**Key points**

**What's known?**

- Subclinical hypomania is common among adolescents and is associated with psychopathological symptoms.
- Little is known about its association and aetiological overlap with psychiatric and neurodevelopmental *diagnoses*.

**What's new?**

- Extending previous research, this study found that subclinical hypomania was significantly associated with 14 phenotypes. The highest odds were for psychotic disorders.
- This is the first study to find small to moderate genetic correlations between subclinical hypomania and 11 diagnoses; the highest was for drug misuse disorders. Small to moderate nonshared environmental correlations were observed for eight diagnoses; the highest was for psychotic disorders.

**What's relevant?**

- These results highlight subclinical hypomania's important role in the developmental pathway to clinical disorders, indicating it could be a plausible prevention and intervention target.

## Supporting information


**Table S1.** Diagnostic classifications for each condition.
**Table S2.** ACE fit model statistics.

## Data Availability

We are unable to share data due to Swedish laws. Researchers wishing to access the data themselves can apply directly to the Swedish Twin Registry.
