# Supplementary material for: Subclinical hypomania, psychiatric and neurodevelopmental diagnoses: phenotypic and aetiological overlap
Source: J Child Psychol Psychiatry. 2025 Sep 6;67(5):686–95. doi: 10.1111/jcpp.70045 (PMC13102046; doi:10.1111/jcpp.70045)
Supplement: Supplementary file 1 — Table S1. Diagnostic classifications for each condition. Table S2. ACE fit model statistics. [file JCPP-67-686-s001.docx]

**Supporting information**

Manuscript entitled: Subclinical hypomania, psychiatric & neurodevelopmental diagnoses: phenotypic & aetiological overlap

**Table S1.** Diagnostic classifications for each condition

**Table S2.** ACE fit model statistics

**Table S1.** Diagnostic classifications for each condition

|  | **Diagnostic classification**  International Classification of Disease-10 (ICD-10) diagnostic codes and Anatomical Therapeutic Chemical Classification System (ATC) codes for medical prescriptions |
| --- | --- |
|  |  |
| Bipolar disorder | F30-F31 + lithium prescription |
| Psychotic disorders | F20 F21-F25, F28-F29 + prescription for Clozapine |
| Depressive disorders | F32-F34 |
| Anxiety disorders | F40-F41 |
| Alcohol use disorders | F10.1-F10.9 |
| Substance use disorders |  |
| Anorexia nervosa | F50.0, F50.1 |
| Other eating disorders | F50.2, F50.3, and F50.9 |
| Borderline personality disorder | F60.3 |
| Obsessive compulsive disorder | F42 |
| ADHD | F90 |
| Autism Spectrum Disorder | F84 |
| Self-harm | X60-X84 Y87.0 Y10-Y34 Y87.2 |
| Sleep disorders and disturbances | F51, G47, prescription of Zaleplon (up until 2012) [N05CF03], Melatonin [N05CH01], Zopiclone [N05CF01] or Zolpidem [N05CF02] |

Abbreviations: ADHD, attention deficit/hyperactivity disorder

**Table S2.** ACE fit model statistics

| **Outcome** | **Model** | **-2LL** | **Parameters** | **df** | **ΔΧ^2^** | **Δdf** | **p** |
| --- | --- | --- | --- | --- | --- | --- | --- |
| Bipolar Disorder | Saturated Model | 27934.39 | 15 | 49542 | ----- | ----- | ----- |
|  | ACE Model | 27951.49 | 11 | 49548 | 17.10 | 6 | 0.009 |
| ADHD | Saturated Model | 37397.20 | 15 | 49542 | ----- | ----- | ----- |
|  | ACE Model | 37441.87 | 11 | 49548 | 44.67 | 6 | 5.452E-08 |
| Alcohol Misuse | Saturated Model | 31603.73 | 15 | 49542 | ----- | ----- | ----- |
|  | ACE Model | 31612.67 | 11 | 49548 | 8.94 | 6 | 0.177 |
| Anorexia Nervosa | Saturated Model | 28804.42 | 15 | 49542 | ----- | ----- | ----- |
|  | ACE Model | 28857.76 | 11 | 49548 | 53.34 | 6 | 1.002E-09 |
| Anxiety Disorders | Saturated Model | 30015.62 | 15 | 49542 | ----- | ----- | ----- |
|  | ACE Model | 30028.27 | 11 | 49548 | 12.65 | 6 | 0.049 |
| Autism | Saturated Model | 29125.36 | 15 | 49542 | ----- | ----- | ----- |
|  | ACE Model | 29141.91 | 11 | 49548 | 16.54 | 6 | 0.011 |
| Depression | Saturated Model | 34475.56 | 15 | 49542 | ----- | ----- | ----- |
|  | ACE Model | 34512.15 | 11 | 49548 | 36.59 | 6 | 2.117E-06 |
| Substance Misuse | Saturated Model | 33245.71 | 15 | 49542 | ----- | ----- | ----- |
|  | ACE Model | 33254.7 | 11 | 49548 | 9.16 | 6 | 0.165 |
| Sleep Difficulties | Saturated Model | 34571.27 | 15 | 49542 | ----- | ----- | ----- |
|  | ACE Model | 34584.58 | 11 | 49548 | 13.30 | 6 | 0.038 |
| OCD | Saturated Model | 28670.74 | 15 | 49542 | ----- | ----- | ----- |
|  | ACE Model | 28678.05 | 11 | 49548 | 7.31 | 6 | 0.293 |
| Other Eating Disorders | Saturated Model | 29349.83 | 15 | 49542 | ----- | ----- | ----- |
|  | ACE Model | 29390.18 | 11 | 49548 | 40.35 | 6 | 3.893E-07 |
| Psychotic Disorders | Saturated Model | 27693.06 | 15 | 49542 | ----- | ----- | ----- |
|  | ACE Model | 27708.34 | 11 | 49548 | 15.28 | 6 | 0.018 |

Abbreviations: ADHD, attention deficit/hyperactivity disorder; OCD, obsessive compulsive disorder
